# Chorea, convulsive seizures and cognitive impairment in a patient with autoimmune thyroid disease

**DOI:** 10.1007/s10072-012-0990-4

**Published:** 2012-03-09

**Authors:** Anna Kamińska, Michał Kamiński, Magdalena Nowaczewska, Barbara Książkiewicz, Roman Junik

**Affiliations:** 1 Department of Endocrinology and Diabetology, Ludwik Rydygier Collegium Medicum in Bydgoszcz, Nicolas Copernicus University in Torun, ul. M. Skłodowskiej-Curie 9, 85-094 Bydgoszcz, Poland; 2 Outpatient’s Clinic of Endocrinology, Oncology Centre in Bydgoszcz, ul. Romanowskiej 2, 85-796 Bydgoszcz, Poland; 3 Department of Neurology, Ludwik Rydygier Collegium Medicum in Bydgoszcz, Nicolaus Copernicus University in Torun, ul. M. Skłodowskiej-Curie 9, 85-094 Bydgoszcz, Poland

Chorea and convulsive seizures are rare consequences of hyperthyroidism [1, 2]. We describe the case of such manifestation of autoimmune thyroid disease.

In 2003, a 23-year-old woman presented with the first symptoms of hyperthyroidism—palpitations, weight loss and exercise intolerance. TSH and fT_4_ concentrations were 0.01 μIU/ml and 43 pmol/l, respectively (norm: TSH 0.35–4.9 μIU/ml, fT_4_ 11.5–23.2 pmol/l). The patient was treated with thiamazole in tapering doses (from 60 to 10 mg per day). After 2 months, hypothyreosis was observed and thiamazole was withdrawn. 2 weeks later, the signs of thyrotoxicosis and involuntary movements of the left extremities appeared. Hyperthyreosis was verified (fT_4_ 87.3 pmol/l) and treatment with propylothiouracil (600 mg per day) was initiated. Neurological examination confirmed left-sided hemichorea. Elevated levels of TSH receptor antibodies (TRAb) and thyroperoxidase autoantibodies (TPO Ab) were documented: 16.7 and 257.5 IU/ml, respectively (norm: 0.1–1.22 IU/ml and <60 IU/ml, respectively). Biopsy specimen of the thyroid showed lymphocytic infiltration, typical of lymphocytic thyroiditis. The patient had no family history of neurological disorders or dementia syndromes. Ceruloplasmin and copper concentrations were normal, similar to anticardiolipin, antistreptolysin O, and anti-neutrophil cytoplasm antibodies. Antinuclear and anti-double-stranded DNA antibodies were negative. Cerebrospinal fluid examination and MRI of the brain were normal, and borreliosis and toxoplasmosis were ruled out based on immunological examination of serum and cerebrospinal fluid. EEG revealed slight changes in the posterior temporal and occipital leads: theta waves and isolated sharp waves, predominantly in the right hemisphere, along with slight changes of paroxysmal character in the frontal and anterior temporal leads. Haloperidol (6 mg per day) and thioridazin (50 mg per day) were administered. fT_4_ concentrations diminished (28.7 pmol/l) and choreic movements considerably subsided.

Treatment with a maintenance dose of thiamazole was continued. Prednisone (60 mg per day, with 5 mg reduction every week) was administered. The dosages of neuroleptics were reduced and finally withdrawn. Choreic movements diminished gradually to disappear completely within 6 weeks. The return of chorea has not been observed so far.

For over a year, during treatment with thiamazole (2.5 mg per day), the patient was euthyroid. In October 2005, in the first trimester of pregnancy, symptoms of thyreotoxicosis were once again reported. TSH and fT_4_ concentrations were 0.02 μIU/ml and 51.73 pmol/l, respectively. Thiamazole was withdrawn and propylothiouracil (initially 150 mg per day) was administered. It was withdrawn in the third trimester, when serum fT_4_ decreased to 14.7 pmol/l. 3 months after the delivery, signs of hyperthyreosis were observed (fT_4_ = 41.7 pmol/l), and treatment with propylothiouracil (150 mg/day) was reinstated. In October 2006, the patient presented with generalized tonic–clonic convulsive seizures, loss of consciousness and retrograde amnesia. fT_3_ and fT_4_ concentrations were elevated (20.11 pg/ml and 5.19 ng/dl, respectively; norm: 1.71–3.71 pg/ml and 0.71–1.85 ng/dl, respectively). Laboratory tests and MRI of the brain were normal. Antithyroid treatment was intensified and the patient became euthyroid in December 2006. TPO Ab and anti-thyroglobulin antibodies (Tg Ab) were elevated (872.7 and 194.8 IU/ml, respectively; norm <60 IU/ml). In June 2008, the patient underwent treatment with radioactive iodine ^131^I (150 MBq).

Between January 2009 and August 2010, the patient was euthyroid and did not receive any pharmacotherapy. During this period of time, she started to suffer from depression, difficulties with her memory, and sleep disturbances. Psychological examination confirmed mild cognitive impairment and mood disorder. Single-photon emission computed tomography of the brain showed slight global hypoperfusion in both cerebral hemispheres, and an unilateral reduction in the regional blood flow of the left parietal lobe, left temporal lobe and left insula. MRI of the brain was normal. EEG revealed slight changes in frontal and temporal leads in the form of theta waves and isolated sharp waves, accompanied by minor paroxysmal features observed in the posterior temporal and occipital leads.

In January 2010, levels of TPO Ab were still elevated (324 IU/ml), while TG Ab and TRAb were normal. In August 2010, subclinical hypothyreosis was diagnosed (TSH 6.13 μIU/ml, fT_4_ 1.36 ng/dl) and treatment with l-thyroxin (25 μg/day) was initiated. Cognitive function deterioration has not been observed thus far.

The pathophysiological mechanism underlying hyperthyroidism-induced chorea is unclear. In most reported cases, choreoathetosis was observed during thyreotoxic states, and one cannot exclude autoimmune mechanisms underlying the chorea [2, 3]. Morphological changes in the basal ganglia are another potential cause of chorea in patients with hyperthyroidism [3]. Seizures are one of the clinical features of encephalopathy associated with autoimmune thyroiditis, often termed Hashimoto’s encephalopathy. The exact pathomechanism of this disease is still not fully understood. Its neurological symptoms can result from autoimmune inflammation of minute cerebral vessels, with the involvement of anti-TPO antibodies [4].

Hereby, presented case illustrates that cognitive impairment may be an unusual consequence of autoimmune thyroid disease, and thyroid function should be examined in subjects with encephalopathy, chorea and seizures.
